# Chemical profile and anti-inflammatory activity of the hydroethanolic extract of *Cochlospermum regium* (Schrank) Pilg. xylopodium

**DOI:** 10.1007/s10787-026-02227-0

**Published:** 2026-04-10

**Authors:** Jessica de Araujo Isaias Muller, Bruna Fioravante Di Serio, Fabiana de Freitas Figueiredo, Marcelo José Dias Silva, Domingos Tabajara de Oliveira Martins

**Affiliations:** 1 Pharmacology Laboratory, Post-Graduate Program in Health Sciences, Federal University of Mato Grosso (UFMT), Cuiabá, MT Brazil; 2 Laboratory of Medicinal Plants and Herbal Medicines, Federal University of Alfenas (UNIFAL-MG), Rua Gabriel Monteiro da Silva, 700, Centro, Alfenas, MG Brazil; 3 Pharmacology Laboratory, Department of Basic Sciences in Health, Federal University of Mato Grosso (UFMT), Cuiabá, MT Brazil

**Keywords:** *Cochlospermum regium*, Phytochemistry, Inflammation, Leukocytes, Cytokines

## Abstract

**Background and aim:**

*Cochlospermum regium* is widely distributed in the Brazilian Cerrado, where its xylopodium is traditionally used to treat inflammatory disorders. This study investigated the anti-inflammatory effects of the hydroethanolic extract of *C. regium* xylopodium (HECr) using in vivo and i*n vitro* models and characterized its phytochemical profile.

**Experimental procedures:**

A 70% hydroethanolic extract was prepared from the xylopodium and subjected to phytochemical analysis. In vivo assays were conducted in male Swiss mice treated with HECr (25, 100, or 400 mg/kg) and evaluated in acetic acid-induced vascular permeability and lipopolysaccharide (LPS)-induced peritonitis models. Total and differential leukocyte counts, and cytokine levels were determined in peritoneal fluid. In vitro, RAW 264.7 macrophages were used to assess cytotoxicity, nitric oxide (NO) production, and inflammatory cytokine release after LPS stimulation.

**Results:**

Phytochemical analysis identified myricetin-3-O-β-D-galactopyranoside, quinic acid, and quercetin-3-O-rhamnoside as major constituents. HECr significantly reduced Evans blue extravasation in the vascular permeability assay. In LPS-induced peritonitis, HECr decreased total leukocyte and neutrophil migration, reduced TNF-α and IL-1β levels, and increased IL-10 concentrations compared with vehicle-treated controls. In LPS-stimulated macrophages, HECr (1–20 µg/mL) reduced IL-1β and NO production, decreased TNF-α at lower concentrations, increased IL-13 levels, and showed no cytotoxicity.

**Conclusions:**

HECr exhibits significant anti-inflammatory activity, likely mediated by modulation of vascular permeability, leukocyte recruitment, cytokines, and NO, supporting its traditional use and potential therapeutic application.

**Graphical abstract:**

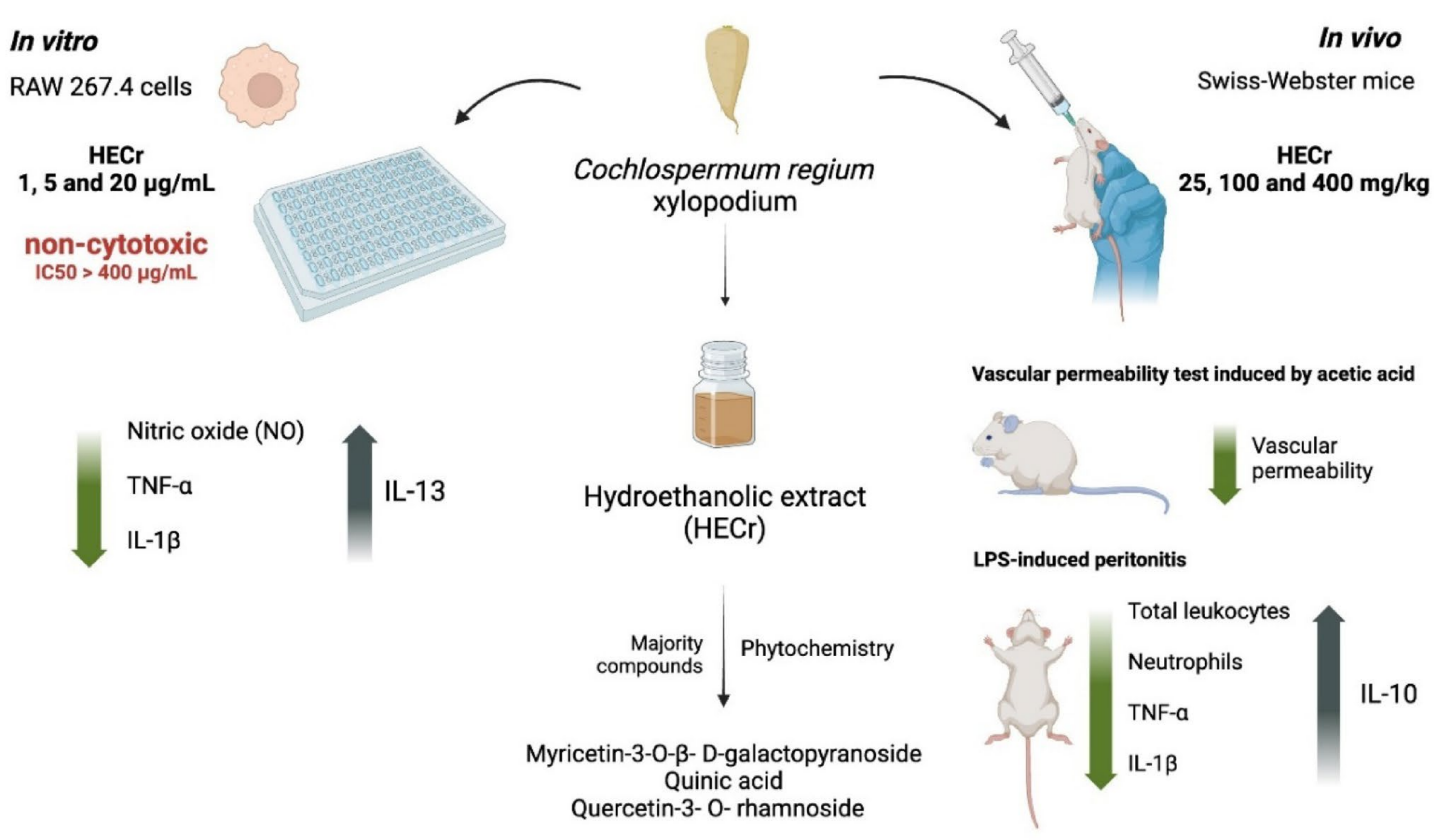

## Introduction

Inflammation is a critical problem in several diseases and conditions. The inflammatory process aims to restore homeostasis after an injury and is necessary for the body. However, if the phlogistic agent is not eliminated or the inflammatory response is exacerbated, it can harm tissues (Yeung et al. 2018) The initial stages of inflammation include the recognition of the damaging agent, which triggers local vascular changes, causing vasodilation, increased vascular permeability, and fluid extravasation (Murphy 2013). which in turn favors the recruitment of inflammatory cells (such as monocytes and neutrophils) to the injured tissue (Kourtzelis et al. 2017). These cells aim to remove harmful agents. If the inflammatory process follows its normal course, the inflammation will resolve, but if it persists for a long time, tissue damage and fibrosis may occur (Kourtzelis et al. 2017; Germolec et al. 2018).

During the inflammatory process, leukocyte cells such as macrophages release mediators, including cytokines and nitric oxide, which act in the different phases of the process (Kumar et al. 2015). These mediators can have both pro- and anti-inflammatory actions, (Holdsworth and Gan 2015) and several medications can act to modulate their release, interfering with important pathways of the inflammatory cascade such as cyclooxygenases (COX), mitogen-activated protein kinase (MAPK), and nuclear factor kappa B (NF-kB) (Yeung et al. 2018).

Non-steroidal anti-inflammatory drugs and corticosteroids remain the most widely used options for treating inflammatory conditions (Brune and Patrignani 2015; Kapugi and Cunningham 2019). Although reducing inflammation may be valuable, these medications may increase the risk of gastrointestinal and coronary events, (Coxib and Traditional NSAID Trialists’ (CNT) Collaboration 2013) and in the case of corticosteroids, weight gain and ulcers may occur, among others (Kapugi and Cunningham 2019). As part of the inflammatory process, pain can be triggered by various inflammatory mediators, and interestingly, medications also have an analgesic action (Omoigui 2007). Therefore, it is necessary to search for new anti-inflammatory drugs that also have the potential to reduce inflammatory pain, with medicinal plants being a possible source (Arulselvan et al. 2016; Tasneem et al. 2019; Gandhi et al. 2022). Among these medicinal species is *Cochlospermum regium* (Schrank) Pilg.

*C. regium* belongs to the Bixaceae family, and its popular names in Brazil include “algodão-do-campo” and “algodão-do-cerrado” (Lieras 2015). This native and non-endemic plant is present in Brazil, Bolivia, and Paraguay (Antar 2018). In Brazilian folk medicine, the xylopodium of *C. regium* is used to treat gastritis; intestinal, uterine, and ovarian inflammations; arthritis; and blackheads; it is also used as a purgative and a menstrual period regulator (Moreira and Guarim-Neto 2009; Ribeiro et al. 2017). In Brazilian folk medicine, *C. regium* xylopodium (underground stem) is traditionally used in the form of slices, chips, or powder to prepare decoctions and infusions (1.35 g/L of water; three cups of tea per day, approximately 600 mL). It is used for the treatment of gastritis, ulcers, and uterine, intestinal, and ovarian inflammations, as well as for blackheads, pimples, and skin blemishes; additionally, it is employed as a purgative, menstrual regulator, depurative, and for the relief of arthritis (Ritto et al. 1996; Moreira and Guarim-Neto 2009; Ribeiro et al. 2017). Regarding its pharmacological properties, ethanolic, hydroethanolic, hydromethanolic, and fluid extracts of the leaves, roots, and xylopodium of *C. regium* have shown anti-inflammatory (Ritto et al. 1996; Galvao et al. 2023), gastroprotective (Arunachalam et al. 2019), antibacterial and antifungal (Almeida-Apolonio et al. 2018; Galvao et al. 2020), antihyperglycemic, anticholinesterase, antioxidant (Pedroso et al. 2019), antinociceptive, and healing activities (Galvao et al. 2023). The hydroethanolic extract of *C. regium* roots showed very low acute toxicity in male and female rats and mice (Toledo et al. 2000). Studies carried out with the hydroethanolic extract of *C. regium* xylopodium (HECr) showed the presence of several secondary metabolites, with emphasis on phenolics, such as gallic acid, myrecitin, and kaempferol (Arunachalam et al. 2019), that may have anti-inflammatory and/or analgesic potential (Bensaad et al. 2017). Despite medicinal plants’ potential for pharmacological use, the correct use of plant material and its regeneration in nature must be considered so that a certain species does not become extinct (Brito 2003). Several studies have evaluated the production capacity of *C. regium in vitro*, aiming to cultivate this species for the production of extracts for medicinal purposes (Gavilan et al. 2018; Inacio et al. 2011). These studies demonstrated that *C. regium* has excellent in vitro germination, making it viable and possible to plant in growth chambers (Camillo et al. 2009), minimizing the environmental impact of using this species.

Therefore, due to the description in the literature of the wide use of *C. regium* in popular medicine, including its use as an anti-inflammatory (Moreira and Guarim-Neto 2009; Ribeiro et al. 2017) and considering the ongoing need for new anti-inflammatory drugs with analgesic potential and the practicality of producing *C. regium in vitro*, this study explored the anti-inflammatory properties of the plant’s hydroethanolic xylopodium extract using in vivo and in vitro assays.

## Materials and methods

### Botanical material processing and extract production

Ten kilograms of *C. regium* xylopodium were collected in Várzea Grande/MT (coordinates: S 15°34′26′′, W 56°14′35′′) in the morning and during the dry season. The plant was authenticated by comparing it with the specimen previously deposited by Arunachalam et al. (2019). The xylopodium (10.5 kg) was cleaned and dried at 40 °C for 72 h, then ground (40 mesh) and macerated for seven days in a 70% hydroethanolic solution (1:10 w/v) at 25 °C, as described by Arunachalam et al. (2019). The HECr obtained (yield = 8.92%) was placed in an amber bottle and stored under refrigeration. For the assays, the extract was resuspended in water or saline according to the test.

The research project was registered on the SisGen Platform (CGen/MMA) to comply with Law 13,123, May 20, 2015, known as the Biodiversity Law (registration number: A7E2B38).

### Drugs and reagents

Dimethyl sulfoxide (DMSO), Dulbecco’s Modified Eagle Medium (DMEM), doxorubicin, dexamethasone acetate (Dexa), resazurin, and lipopolysaccharide from *Escherichia coli* (serotype 055:B5) were sourced from Sigma-Aldrich Co. (USA). Additional reagents included 70% ethanol, sterile 0.9% saline, Evans blue, and trypan blue (Dinâmica Química Contemporânea Ltda., Brazil); ketamine and xylazine (Syntec, Brazil); as well as streptomycin, penicillin, non-essential amino acids, glutamine, fetal bovine serum (FBS), and ELISA kits for TNF-α, IL-1β, IL-10, and IL-13 (Thermo Fisher Scientific, USA). Turk’s solution and Instant Prov^®^ (Quick Panoptic stain) were obtained from New Prov (Brazil).

### Animals

Adult male Swiss-Webster mice (*Mus musculus*), weighing between 20 and 25 g, from the UFMT Central Animal House were used. The animals were kept at the Animal Experimentation Laboratory in polypropylene boxes for acclimatization in an environment at 23 ± 2 °C and with a 12-h light/dark cycle, receiving standard food (Nuvilab^®^, Quimtia, Brazil) and treated tap water *ad libitum*. Before the tests, the animals were fasted for 12 h. The experiments were conducted in accordance with the standards established by the Brazilian College of Animal Experimentation (COBEA) and approved by the UFMT Ethics Committee on the Use of Animals (CEUA; process no. 23108.011151/2022-12). The animals were euthanized using a lethal dose of the anesthetic xylazine (30 mg/kg) and ketamine (300 mg/kg) delivered via intraperitoneal (i.p.) injection (UNIFESP 2009).

### Cell line

This study employed RAW 264.7 murine macrophages (code 0212), acquired from the Rio de Janeiro Cell Bank (BCRJ, Brazil). The cells were cultured in DMEM supplemented with 10% fetal bovine serum and maintained at 37 °C in a humidified atmosphere containing 5% CO₂.

### Phytochemical analysis

The HECr was pretreated in a SPE C18 cartridge (3 mL, Macherey-Nagel, Chromabond C18, Duren, Germany). The sorbent presented a particle dimension of 45 μm,, and the diameter and pore size were 60 Å. The HECr (10 mg) was solubilized in 1.5 mL of methanol: water mixture (MeOH: H_2_O, 80:20, v/v. MeOH: LC-MS grade, LiChrosolv^®^, and H_2_O ultrapure MilliQ^®^ water, Eq. 7000). The elution was done as per Venturini et al. 2024; and the sample was dissolved in MeOH to be evaluated by high-performance liquid chromatography.

### Analysis using direct flow infusion–electrospray ionization ion trap MSⁿ (DFI-ESI-IT-MSⁿ) and ultra-high-performance liquid chromatography–electrospray ionization MSⁿ (UHPLC-ESI-MSⁿ) on the LCQ fleet™ system

The sample was directly introduced into a Thermo Fisher Scientific LTQ XL linear ion trap mass spectrometer equipped with an electrospray ionization (ESI) source operating in negative mode (Thermo Fisher Scientific, San Jose, CA, USA) (DFI-ESI-IT-MSⁿ). A fused-silica capillary was maintained at 300 °C, with a spray voltage of 4.5 kV, a capillary voltage of − 47 V, and a tube lens voltage of − 226 V. Nitrogen (50 arbitrary units) and helium (10 arbitrary units) were used as the nebulizing and collision gases, respectively. Initially, a full mass scan ranging from 50 to 2000 m/z was conducted to stabilize the instrument. Subsequently, MS/MS and UHPLC-ESI-IT-MS analyses were carried out following the methodology described by Dantas-Medeiros et al. 2021.

Chromatographic separation employed a C18 reversed-phase column (Phenomenex^®^ Luna C18, 250 mm × 4.6 mm × 5 μm). The sample (10 µL) was injected by direct infusion at a flow rate of 350 µL min⁻¹ and kept at 25 °C, with UV detection performed at 254 and 354 nm. The elution gradient ranged from 5% to 100% solvent B (acetonitrile/water containing 0.1% formic acid) against solvent A (water with 0.1% formic acid) during60 min. All data capture and processing steps were completed in Xcalibur™ version 1.3 (Thermo Fisher Scientific, San Jose, CA, USA).

### In vivo assays

#### Vascular permeability

The vascular permeability assay was carried out according to the methodology of Whittle (1964). The mice (n = 8/group) received orogastric gavage (p.o.), with vehicle (distilled water 0.1 mL/10 g body weight), three doses of HECr (25, 100, and 400 mg/kg), and dexamethasone (0.5 mg/kg). One group (naive) was treated only with distilled water p.o. and sterile 0.9% saline (i.p.). After 40 min, Evans blue (2% w/v, 0.1 mL/10 g in 0.9% saline) was injected into the penile venous plexus. After 20 min, 0.5 mL of 0.6% (v/v) acetic acid was injected i.p. and 30 min later, mice were euthanized with ketamine/xylazine overdose. The peritoneal cavity was washed with cold phosphate-buffered saline (PBS), and the collected fluid was centrifuged (1000 rpm, 100.8 × g, 10 min). Supernatant absorbance was measured at 620 nm using a spectrophotometer (Thermo Fisher Scientific, USA). The amount of dye extravasated into the peritoneal cavity was then calculated from an Evans blue standard curve (0.78–100 µg/mL) and expressed as µg Evans blue/mL.

#### Lipopolysaccharide-induced inflammatory peritonitis model

The induction of peritonitis was performed according to Souza and Ferreira (1985). Mice (*n* = 8/group) received oral pretreatment with vehicle (distilled water, 0.1 mL/10 g b.w.), three doses of HECr (25, 100, and 400 mg/kg), or dexamethasone (0.5 mg/kg). A naive group received only distilled water (p.o.) and 0.9% sterile saline (i.p.). After 1 h, the mice received an intraperitoneal injection of lipopolysaccharide (LPS) (*Escherichia coli* 055:B5 strain − 250 ng in 0.2 mL of 0.9% sterile saline). 6 h after LPS injection, euthanasia was performed using a lethal i.p. dose of ketamine and xylazine, and peritoneal lavage was collected by washing the peritoneal cavity with 3 mL of ice-cold 0.1% PBS containing 1% bovine serum albumin. The lavage was used for total and differential leukocyte counts, and samples were stored at − 80 °C for cytokine analysis.

##### Leukocyte Total and Differential Counts

An aliquot of the peritoneal lavage was diluted in Turk’s solution at a 1:20 (v/v) ratio and subsequently analyzed using a Neubauer counting chamber under a light microscope (Nikon) at 1000× magnification. A second aliquot was prepared for leukocyte differential counting, in which cells smears were prepared on glass slides and stained with Instant Prov^®^ (New Prov, Brazil). For analysis, 100 cells were examined and categorized as polymorphonuclear (PMN) or mononuclear (MN) according to morphological characteristics. Data were presented as the number of cells × 10⁶.

##### Determination of cytokines in vivo

Cytokine levels (pg/mL) for TNF-α, IL-1β, and IL-10 in the peritoneal lavage were measured using an ELISA kit. (Invitrogen, Austria) following the manufacturer’s recommendations. The results were expressed as pg/mL.

### In vitro assays

#### Cytotoxicity assay on RAW 264.7 cells

The methodology used was the resazurin test (Nakayama et al. 1997). RAW 264.7 cells (2 × 10⁴ cells/mL) were allocated in 96-well plates (duplicate) and incubated overnight at 37 °C with 5% CO₂ for adhesion. The experimental groups were: (1) cells + culture medium; (2) cells and LPS (1 µg/mL, serotype 0127:B8); (3) cells + LPS + HECr (6.25–400 µg/mL); (4) cells + HECr (6.25–400 µg/mL, without LPS); and (5) cells + doxorubicin (100–0.01 µM). After 24, 48, and 72 h, the medium was replaced with 200 µL resazurin (10% v/v) and plates were incubated for 6 h (37 °C, 5% CO₂). Absorbance was measured at 540 and 620 nm. The results were reported as the concentration that inhibited 50% of cell proliferation (IC_50_), considering cytotoxic IC_50_ values of < 4 µg/mL for pure drugs and < 30 µg/mL for impure drugs (Suffness and Pezzuto 1990).

#### Determination of nitric oxide production

Nitric oxide (NO) production was indirectly assessed by measuring nitrite (NO₂⁻) using the Griess reaction (Park et al. 2011). Cells were treated with three concentrations of HECr (1, 5, and 20 µg/mL) that had shown no cytotoxicity in the resazurin assay. The positive control consisted of Nω-nitro-L-arginine-methyl-ester (L-NAME, 10 mM) applied for 1 h prior to LPS stimulation (1 µg/mL). Additional controls included cells maintained in culture medium alone or in medium with LPS. After 24 h, 100 µL of the supernatant was mixed with an equal volume of Griess reagent (1% sulfanilamide, 0.1% N-(1-naphthyl)-ethylenediamine dihydrochloride, 2.5% H₃PO₄) and incubated at room temperature for 15 min. Absorbance was measured at 540 nm using a microplate spectrophotometer, and NO₂⁻ concentrations were calculated from a sodium nitrite (NaNO₂) standard curve, expressed in µM, to estimate NO levels.

#### Determination of cytokines

The levels of TNF-α, IL-1β, and IL-13 (pg/mL) were measured in the supernatants of RAW 264.7 cells stimulated with LPS (1 µg/mL) and treated with three concentrations of HECr (1, 5, and 20 µg/mL). Control groups included cells maintained in culture medium alone or in culture medium with LPS. After 24 h, the supernatants were collected, and cytokine concentrations were determined using ELISA kits (eBioscience^®^, San Diego, USA) according to the manufacturer’s instructions.

### Statistical analysis

Parametric data are presented as mean ± standard error (S.E.). Comparisons among more than two groups were conducted using one-way analysis of variance (ANOVA). When significant differences were observed, the Student–Newman–Keuls post hoc test was applied. Statistical significance was defined as *p* < 0.05.

IC₅₀ values were obtained by linear regression of the inhibition percentage plotted against the logarithm of the tested concentrations, using a 99% confidence level (*p* < 0.01). Data organization and statistical analyses were executed with GraphPad Prism software (v5.01).

## Results

### Phytochemical analysis of HECr

**Fig. 1:**
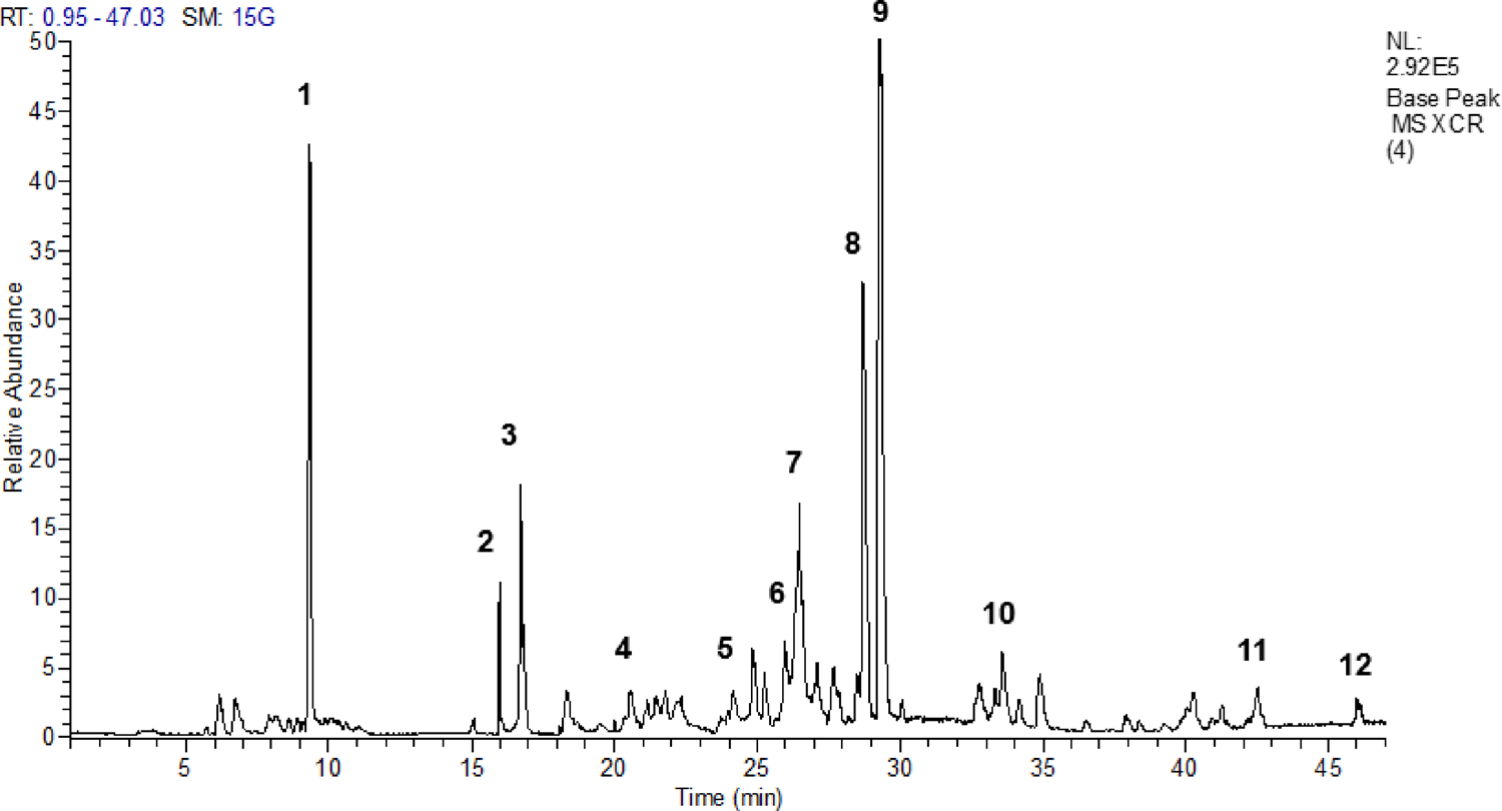
UHPLC-ESI-IT-MSⁿ analysis of the hydroethanolic extract of *Cochlospermum regium* xylopodium was performed with detection at 254 and 354 nm. Chromatographic profiles were compared with reference standards. Peaks of interest, with a signal match value of 1.86 × 10⁵, correspond to the compounds listed in Table 1

The UHPLC-MS chromatographic profile of the extract showed 12 compounds: (1) quinic acid, (2) ellagic acid glucuronide, (3) galloyl glucose, (4) galloyl hexahydroxydiphenoyl hexoside, (5) trigalloyl glucose, (6) digalloyl-glucoside, (7) dihydrokaempferol rhamnoside, (8) quercetin-3-O-rhamnoside, (9) myricetin-3-O-β-D-galactopyranoside, (10) ellagic acid, 11) luteolin 7-O-[6’’-dihydrogalloyl]-glucosyl-8-C-pentosyl-(1→2)-glucoside, and 12) quercetin-7-O-glucoside, as shown in Fig. 1; Table 1.

**Table 1:** Compounds identified from fragmentation pattern analysis obtained using both UHPLC-ESI-IT-MSⁿ and DFI-ESI-IT-MSⁿ in the hydroethanolic extract of *Cochlospermum regium* xylopodium

*N*°	T_*r* (min)_	[M-H]^−^	MS/MS	Compound
1	9.33	191	127, 85, 57	Quinic acid
2	15.98	477	301	Ellagic acid glucuronide
3	16.74	331	271	Galloyl glucose
4	20.54	633	301 463 275 481	Galloyl hexahydroxydiphenoyl hexoside
5	24.16	635	465 313 169 125	Trigalloyl glucose
6	25.99	483	271,211, 193,169, 125	Digalloyl-glucoside
7	26.47	449	287	Dihydrokaempepferol rhamnoside
8	28.72	447	301, 300, 273, 229, 151	Quercetin-3- O- rhamnoside
9	29.30	479	316, 271, 24	Myricetin-3-O-β- D-galactopyranoside
10	33.56	300	284, 229, 185, 145, 129	Ellegic acid
11	42.54	895	563, 447, 357, 339, 327, 285	Luteolin 7-O-[6”-dihydrogalloyl]-glusosyl-8-C-pentosyl-1(1→2)-glucoside
12	46.00	463	301, 300, 179, 151, 149	Quercetin-7- O-glucoside

### In vivo assays

#### Effect of HECr on reducing vascular permeability

**Fig. 2:**
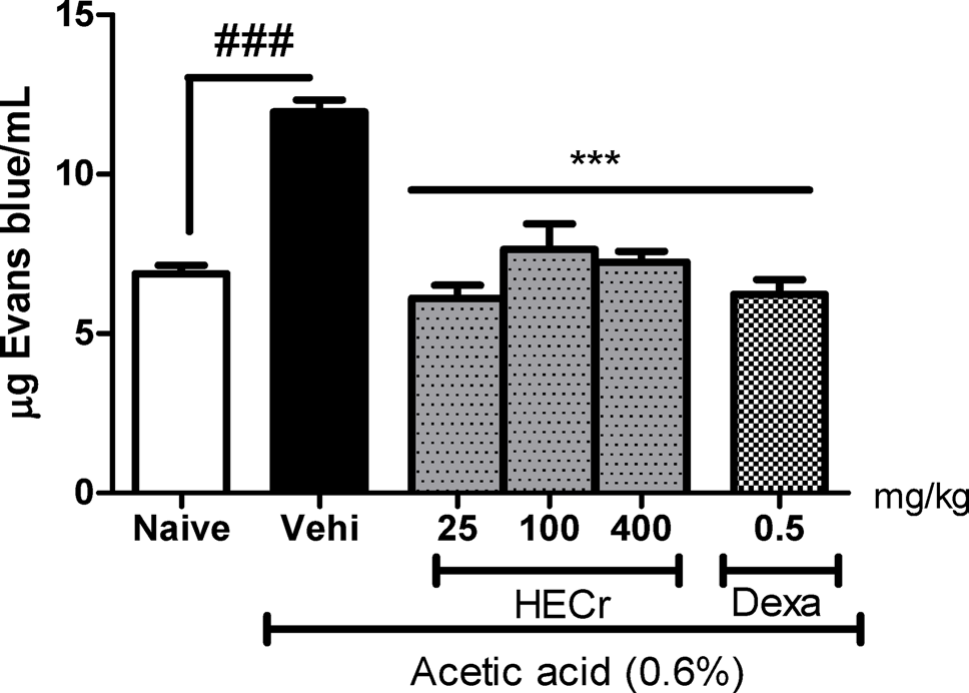
Effect of the vehicle (Vehi, 0.1 mL/10 g of sterile distilled water, p.o.), hydroethanolic extract of *Cochlospermum regium* xylopodium (HECr, 25, 100, and 400 mg/kg, p.o.), and dexamethasone (Dexa, 0.5 mg/kg, p.o.) on acetic acid-induced increases in peritoneal vascular permeability in mice. The naive group received only distilled water (p.o.) and 0.9% sterile saline (i.p.). Data are presented as mean ± S.E. for 8 animals per group. Statistical analysis was performed using one-way ANOVA followed by the Student–Newman–Keuls test. *** *p* < 0.001 vs. Vehi, ^###^ *p* < 0.001 vs. Naive

In vascular permeability induced by acetic acid (0.6%), the vehicle group (distilled water 0.1 mL/10 g p.o.) showed an increase of 73.8% (*p* < 0.001) in vascular permeability relative to the naive group. Treatment with HECr at doses of 25, 100, and 400 mg/kg reduced (*p* < 0.001) the extravasation of Evans blue into the peritoneal cavity by 48.9%, 36.1%, and 39.5%, respectively. Dexamethasone (0.5 mg/kg), the positive control, reduced vascular permeability by 47.9% (*p* < 0.001) (Fig. 2).

#### Effect of HECr on LPS-mediated peritoneal inflammation

**Fig. 3:**
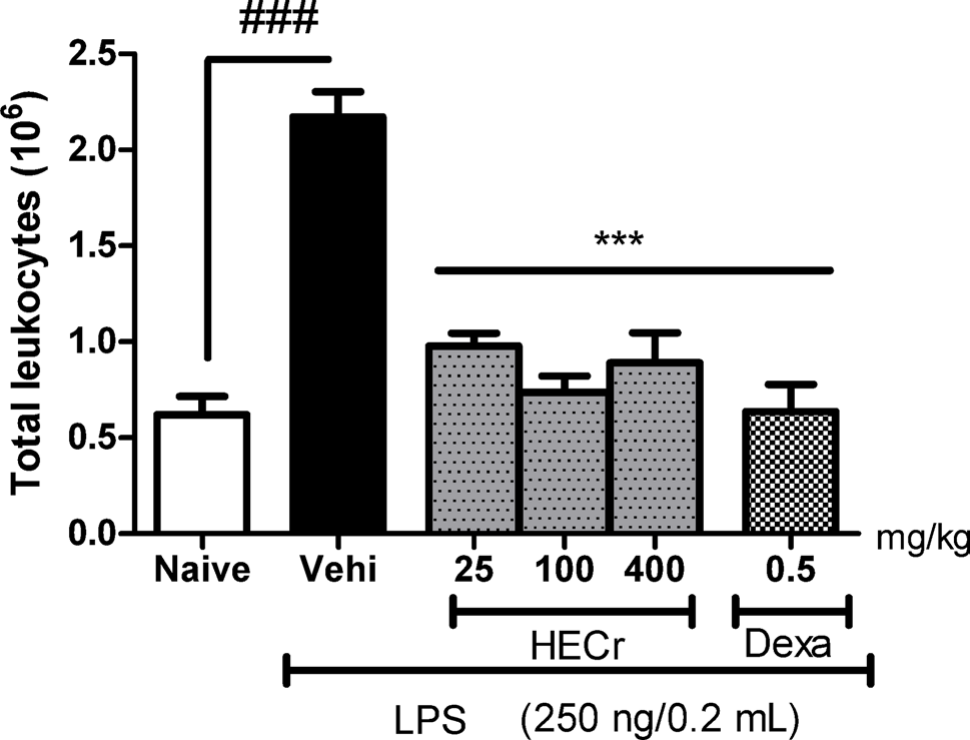
Effect of the vehicle (Vehi, 0.1 mL/10 g b.w. of sterile distilled water, p.o.), hydroethanolic extract of *Cochlospermum regium* xylopodium (HECr, 25, 100, and 400 mg/kg, p.o.), and dexamethasone (Dexa, 0.5 mg/kg, p.o.) on the total leukocyte count following LPS injection (250 ng/0.2 mL/well, i.p.). The naive group received only distilled water (p.o.) and 0.9% sterile saline (i.p.). Data are expressed as mean ± S.E. for 8 animals per group. Statistical analysis was performed using one-way ANOVA with Student–Newman–Keuls post hoc test. *** *p* < 0.001 vs. Vehi, ^###^ *p* < 0.001 vs. Naive

In peritonitis induced by LPS (250 ng/0.2 mL), the vehicle group (distilled water 0.1 mL/10 g p.o.) showed an increase of 250.2% (*p* < 0.001) in the total number of cells in the peritoneal lavage relative to the naive group. Treatment with HECr at doses of 25, 100, and 400 mg/kg reduced (*p* < 0.001) the number of total leukocytes by 54.9%, 66.1%, and 58.9%, respectively. Dexamethasone (0.5 mg/kg) decreased the total cell count by 70.7% (*p* < 0.001) (Fig. 3).

**Fig. 4:**
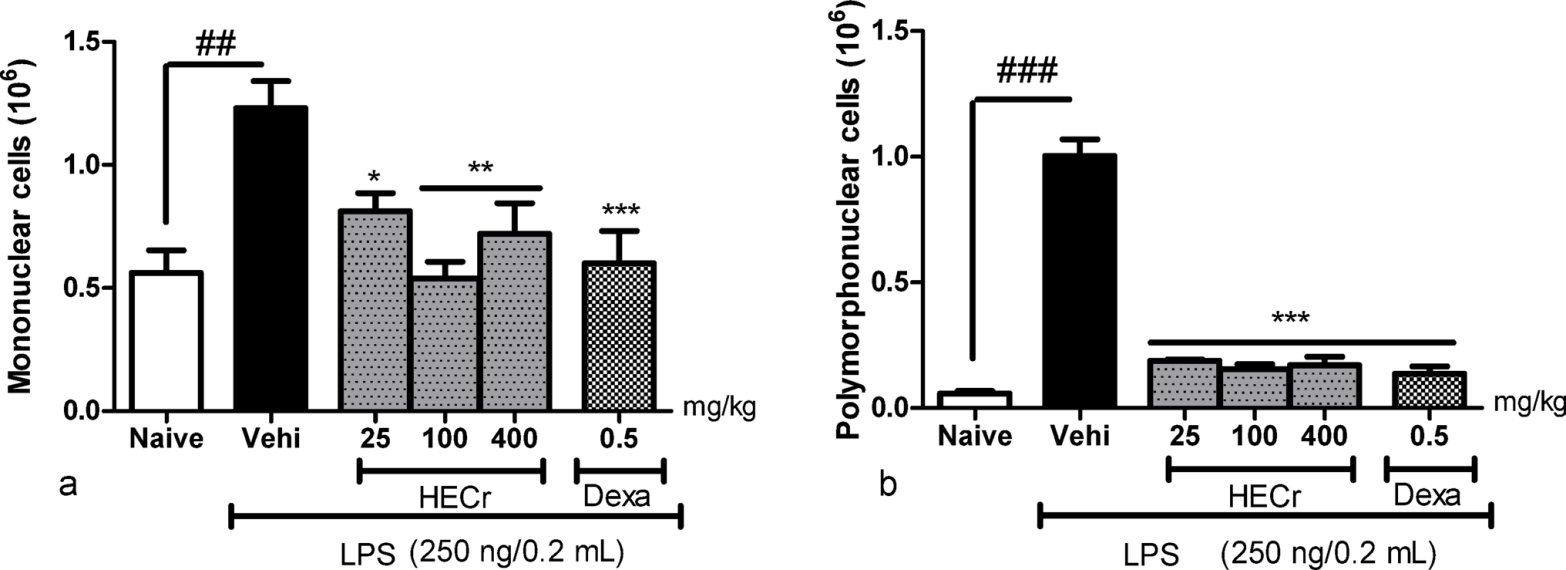
Effect of the vehicle (Vehi; 0.1 mL/10 g sterile distilled water, p.o.), the hydroethanolic extract of *Cochlospermum regium* xylopodium (HECr; 25, 100, and 400 mg/kg, p.o.), and dexamethasone (Dexa, 0.5 mg/kg, p.o.) on the number of mononuclear (**A**) and polymorphonuclear (**B**) cells in the peritoneal lavage of mice with LPS-induced peritonitis (250 ng in 0.2 mL, i.p.). The naïve group received distilled water (0.1 mL/10 g, p.o.) and 0.9% saline (0.2 mL, i.p.). Values represent the mean ± S.E. for eight animals per group. Data were analyzed by one-way ANOVA with Student–Newman–Keuls post hoc test. *p < 0.05, **p < 0.01 and *** *p* < 0.001 vs. Vehi; ^##^ *p* < 0.001 vs. naive

In the evaluation of MN cells, after LPS induction, the vehicle group (distilled water 0.1 mL/10 g b.w.) showed an increase of 118.9% (*p* < 0.01) in the number of MN cells in the peritoneal lavage relative to naïve group. Treatment with HECr at doses of 25, 100, and 400 mg/kg reduced the number of MN cells by 34.0% (*p* < 0.05), 56.3% (*p* < 0.01), and 41.4% (*p* < 0.01), respectively. The standard positive-control treatment with dexamethasone (0.5 mg/kg) resulted in a 51.1% reduction in the number of these cells (*p* < 0.001; Fig. 4A).

In the evaluation of PMN cells, after induction by LPS, the vehicle group (distilled water 0.1 mL/10 g b.w.) showed an increase of 1629.3% (*p* < 0.001) in the number of PMN cells in the peritoneal lavage compared to the naive group. Treatment with HECr at doses of 25, 100, and 400 mg/kg reduced (*p* < 0.001) the number of PMN cells by 81.3%, 84.7% and 83.0%, respectively. Dexamethasone (0.5 mg/kg) reduced PMN cell counts by 86.4% (*p* < 0.001) (Fig. 4B). All the effects of the HECr on MN and PMN cell counts were not dose-dependent.

#### Effect of HECr on cytokine production

**Fig. 5:**
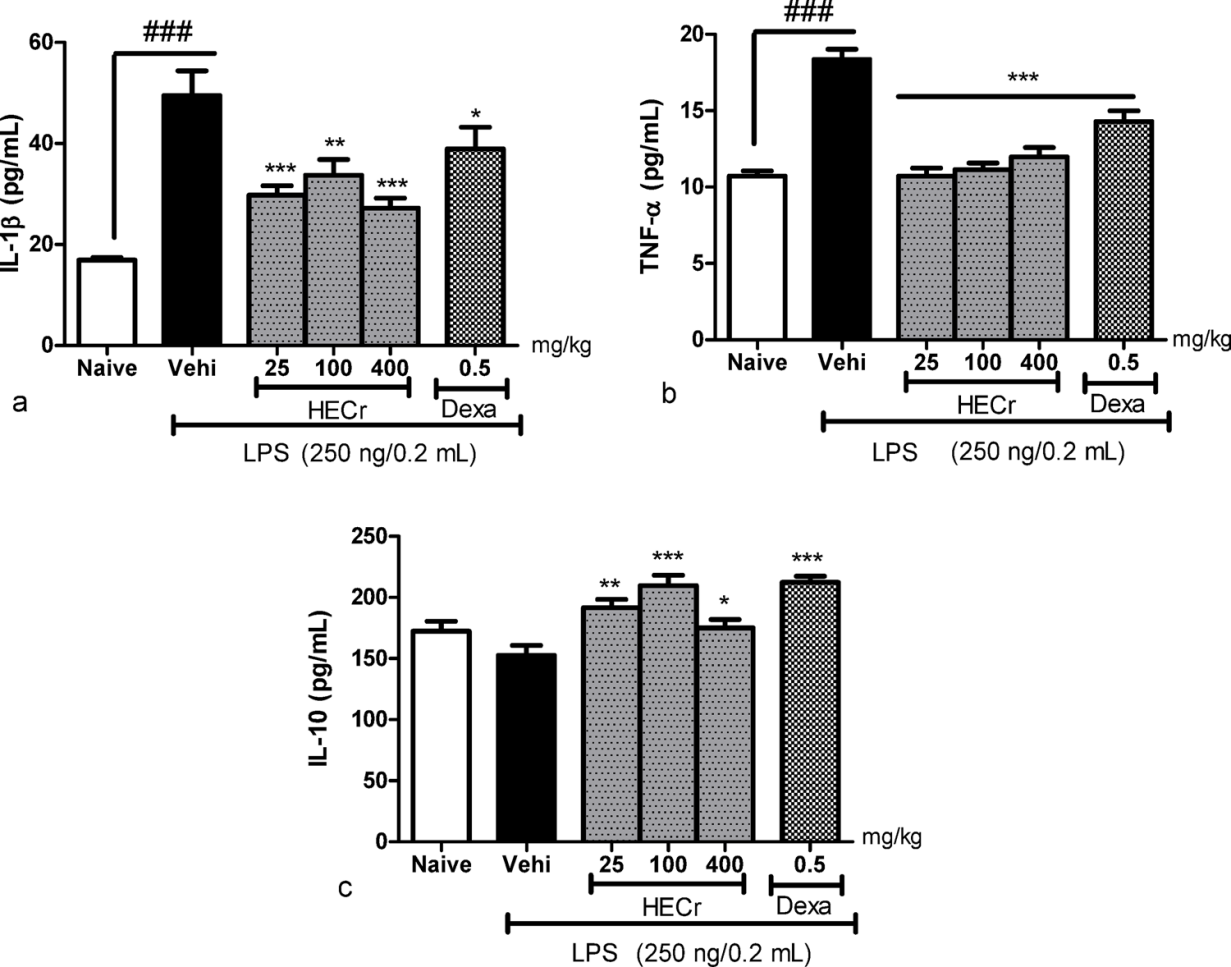
Effect of the vehicle (Vehi; 0.1 mL/10 g sterile distilled water, p.o.), the hydroethanolic extract of *Cochlospermum regium* xylopodium (HECr; 25, 100, and 400 mg/kg, p.o.), and dexamethasone (Dexa; 0.5 mg/kg, p.o.) on the levels of (**A**) interleukin-1β (IL-1β), (**B**) tumor necrosis factor-α (TNF-α), and (C) IL-10 in the peritoneal lavage of mice with LPS-induced peritonitis (250 ng/well in 0.2 mL, i.p.). The naïve group received distilled water (0.1 mL/10 g, p.o.) and 0.9% saline (0.2 mL, i.p.). Values represent the mean ± S.E. for eight animals per group. Data were analyzed by one-way ANOVA with Student–Newman–Keuls post hoc test. * *p* < 0.05, ** *p* < 0.01 and *** *p* < 0.001 vs. Vehi,^###^ *p* < 0.001 vs. naive

In the LPS-induced peritonitis model, the vehicle group (distilled water, 0.1 mL/10 g, p.o.) exhibited a 192.2% increase in IL-1β levels in peritoneal lavage relative to the naive group (*p* < 0.001). Administration of HECr at doses of 25, 100, and 400 mg/kg led to a reduction in IL-1β levels by 39.8% (*p* < 0.001), 31.9% (*p* < 0.01), and 45.0% (*p* < 0.001), respectively, relative to the vehicle group. Dexamethasone (0.5 mg/kg) decreased IL-1β by 21.4% (*p* < 0.05) (Fig. 5A).

For TNF-α, LPS-induced peritonitis increased cytokine levels in peritoneal lavage by 71.1% compared to the naive group (*p* < 0.001). HECr treatment at 25, 100, and 400 mg/kg significantly reduced TNF-α levels by 41.6%, 39.3%, and 34.7%, respectively (*p* < 0.001). Dexamethasone (0.5 mg/kg) also reduced TNF-α by 22.2% (*p* < 0.001) compared to the vehicle group (Fig. 5B).

Regarding the anti-inflammatory cytokine IL-10, no difference was observed between the vehicle and naive groups in LPS-induced peritonitis. Treatment with HECr at 25, 100, and 400 mg/kg increased IL-10 levels by 25.5% (*p* < 0.01), 37.4% (*p* < 0.001), and 14.7% (*p* < 0.05), respectively, relative to the vehicle group. The positive-control group treated with dexamethasone (0.5 mg/kg) exhibited a 39.1% increase in IL-10 levels (*p* < 0.05) (Fig. 5C). Notably, the effects of HECr on cytokine production were not dose-dependent.

### In vitro assays

#### Effect of HECr on cell viability

**Fig. 6:**
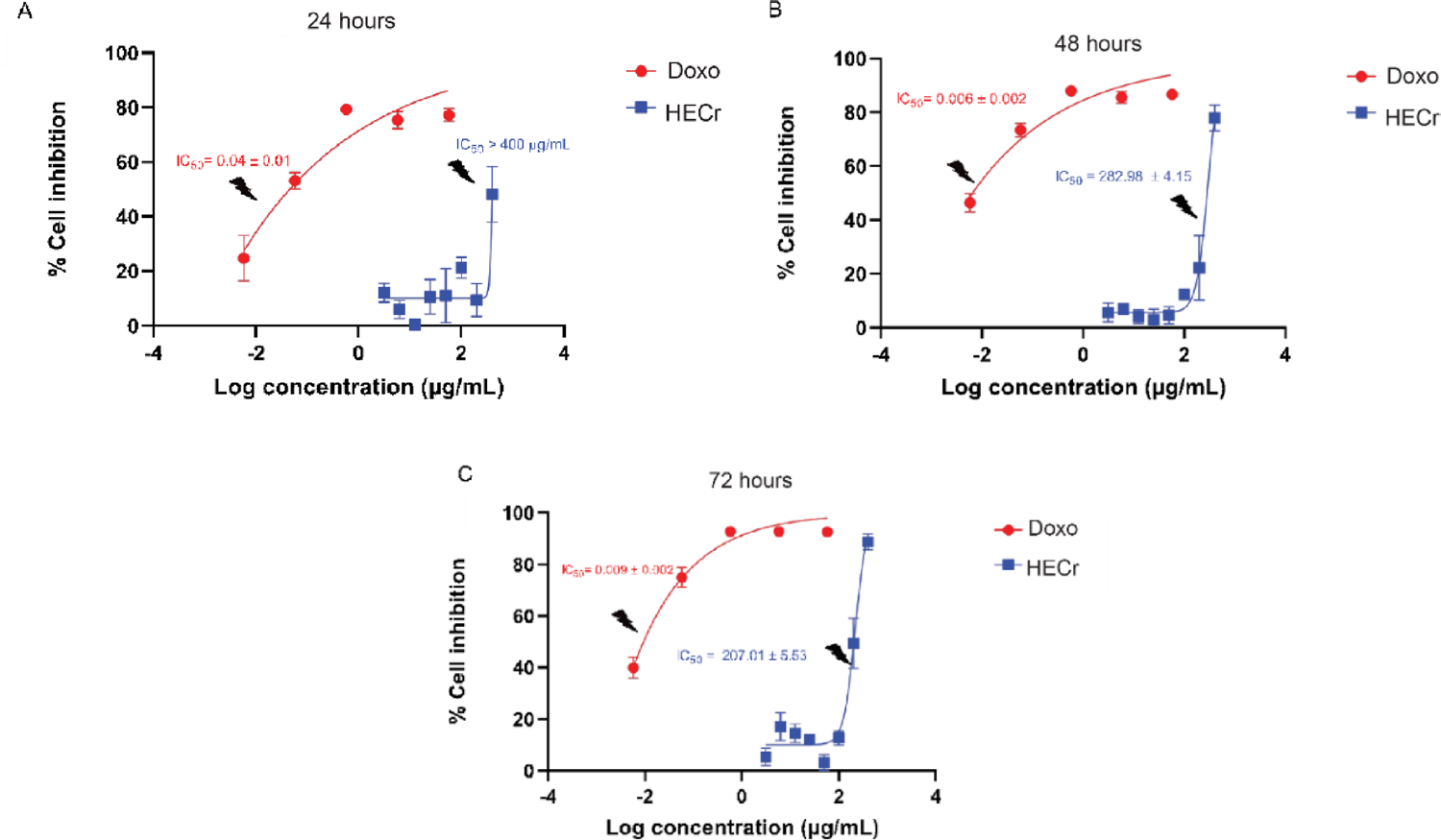
Cytotoxicity assessment of the hydroethanolic extract of *Cochlospermum regium* xylopodium (HECr, 6.25–400 µg/mL) and doxorubicin (0.01–100 µM) in LPS-stimulated RAW 264.7 cells (1 µg/mL) evaluated at 24 (**A**), 48 (**B**), and 72 h (**C**). Data are expressed as the inhibitory concentration at 50% (IC₅₀ ± S.E.) and were determined by nonlinear regression (curve fitting)

No cytotoxic effect was observed for HECr at 24 h (IC_50_ > 400 µg/mL), 48 h (IC_50_ = 282.98 ± 4.15 µg/mL), and 72 h (IC_50_ = 207.01 ± 5.53 µg/mL) in the assays with RAW 264.7 cells. The positive-control compound, doxorubicin, demonstrated toxicity after 24 h, with IC_50_ = 0.04 ± 0.01 µg/mL (Fig. 6A), while at 48 and 72 h, it presented even greater cytotoxicity, with IC_50_ = 0.006 ± 0.002 µg/mL (Fig. 6B) and IC_50_ = 0.009 ± 0.002 µg/mL (Fig. 6C), respectively. LPS did not show cytotoxicity at the concentration (1 µg/mL) used to stimulate RAW 264.7 cells (data not shown).

#### Effect of HECr on nitric oxide production

**Fig. 7:**
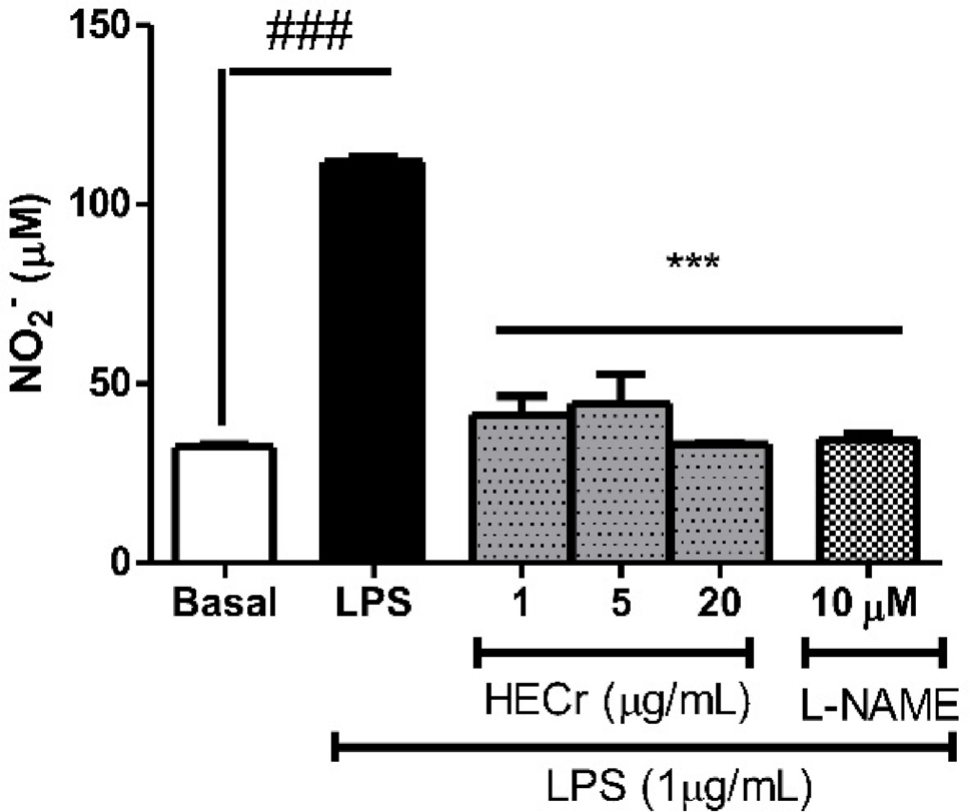
Effect of hydroethanolic extract of *Cochlospermum regium* xylopodium (HECr 1, 5, and 20 µg/mL) and Nω-nitro-L-arginine methyl ester hydrochloride (L-NAME 10 mM) on nitric oxide (NO) production [measured by indirect determination of nitrite (NO_2_^−^)] in LPS-stimulated RAW 264.7 cells (1 µg/mL). The basal group contained only the medium and cells and did not receive LPS. One-way ANOVA, followed by the Student-Newman-Keuls test, *** *p* < 0.001 vs. LPS, ^###^ *p* < 0.001 vs. Basal

When measuring indirect NO concentration in RAW 264.7 cells stimulated with LPS (1 µg/mL) and untreated with HECr, the cell supernatant showed a 245.2% elevation in NO levels (*p* < 0.001) relative to baseline. Treatment with HECr at concentrations of 1, 5, and 20 µg/mL reduced NO levels by 62.9%, 60.2%, and 70.3% (*p* < 0.001), respectively, when compared to the vehicle group. In cells treated with L-NAME (10 mM), the NO level reduction was 69.16% (*p* < 0.001) relative to the vehicle group (Fig. 7).

#### Effect of HECr on cytokine production

**Fig. 8:**
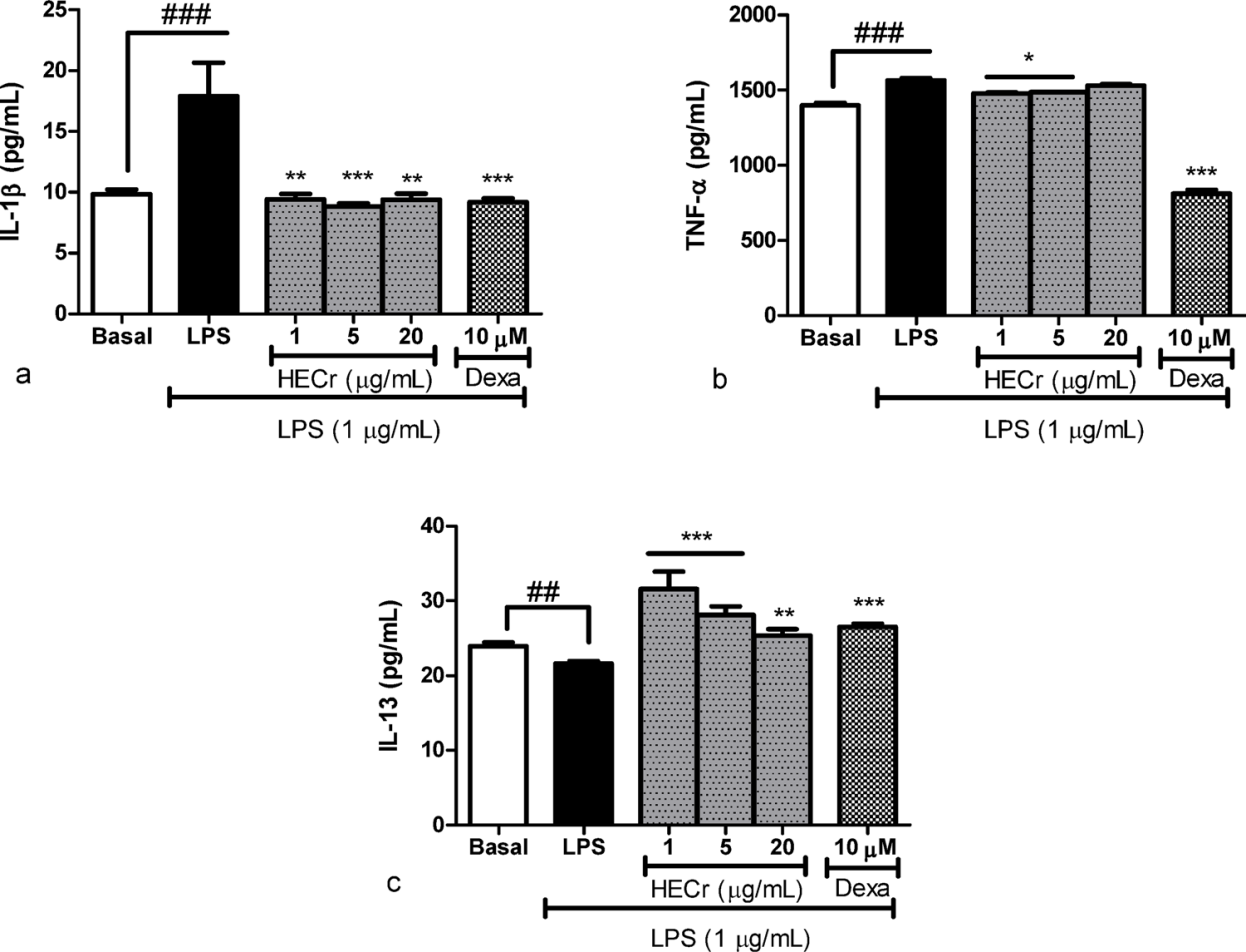
Effect of the hydroethanolic extract of *Cochlospermum regium* xylopodium (HECr; 1, 5, and 20 µg/mL) and dexamethasone (Dexa; 10 µM) on the levels of (**A**) interleukin-1β (IL-1β), (**B**) tumor necrosis factor-α (TNF-α), and (**C**) IL-13 in RAW 264.7 cells stimulated with LPS (1 µg/mL). The basal group did not receive LPS. Data were analyzed using one-way ANOVA followed by the Student–Newman–Keuls test. * *p* < 0.05, ** *p* < 0.01 and *** *p* < 0.001 vs. LPS, ^###^ *p* < 0.001 vs. Basal

RAW 264.7 cells were stimulated with LPS (1 µg/mL), and the untreated group showed an increase of 81.8% (*p* < 0.001) in the level of IL-1β relative to baseline. Treatment with HECr at 1, 5, and 20 µg/mL reduced the levels of IL-1β by 47.3% (*p* < 0.01), 50.7% (*p* < 0.001), and 47.4% (*p* < 0.01), respectively, when compared to the LPS group. Dexamethasone (10 µM) reduced it by 48.6% (*p* < 0.001) (Fig. 8A).

In the quantification of TNF-α, RAW 264.7 macrophages stimulated with LPS (1 µg/mL) showed an increase of 11.9% (*p* < 0.001) in the level of TNF-α relative to baseline. Treatment with HECr at concentrations of 1 and 5 µg/mL reduced TNF-α levels by 5.5% and 4.9% (*p* < 0.05), respectively, when compared to the LPS group. Dexamethasone (10 µM) reduced it by 48.1% (*p* < 0.001) (Fig. 8B).

In the assay with the anti-inflammatory cytokine IL-13, RAW 264.7 cells were stimulated with LPS (1 µg/mL), and the untreated group showed a 9.8% reduction (*p* < 0.01) relative to baseline. The administration of HECr at concentrations of 1, 5, and 20 µg/mL increased the levels of IL-13 by 46.1%, 30.1% (*p* < 0.001), and 17.4% (*p* < 0.01), respectively, compared to the LPS group. Dexamethasone (10 µM) increased it by 22.7% (*p* < 0.001) (Fig. 8C).

## Discussion

The present findings substantiate the traditional use of *C. regium* xylopodium in inflammatory conditions (Moreira and Guarim-Neto 2009; Ribeiro et al. 2017). The hydroethanolic extraction was chosen because it allows for better preservation of the extracted material and is more efficient for obtaining active pharmacological constituents than aqueous extracts, which are more susceptible to microbial growth and oxidation (Rosa et al. 2017). Furthermore, hydroethanolic solutions in plant extract preparations constitute one of the forms recognized by the current Brazilian Pharmacopeia (Brasil 2024). The doses used (25, 100, and 400 mg/kg, p.o.) in the in vivo anti-inflammatory assays were based on previous studies by Arunachalam et al. (2019) and on the toxicity studies conducted by Toledo et al. (2000).

The hydroethanolic extract (HECr) demonstrated significant anti-inflammatory activity in vivo by reducing vascular permeability, leukocyte migration — particularly neutrophils — and pro-inflammatory cytokines (TNF-α and IL-1β), while increasing the anti-inflammatory cytokine IL-10. The reduction in Evans blue extravasation indicates modulation of early vascular events, which are driven by mediators such as histamine, prostaglandins, leukotrienes, and cytokines (Almeida et al. 2016; Bont et al. 2006; McDonald et al. 2010). The decrease in TNF-α and IL-1β likely contributed to diminished endothelial activation and neutrophil recruitment, consistent with the marked inhibition (> 80%) of neutrophil influx in LPS-induced peritonitis.

In vitro assays using RAW 264.7 macrophages confirmed these effects and excluded relevant cytotoxicity. HECr markedly inhibited nitric oxide (NO) production, probably via modulation of inducible nitric oxide synthase activity. Since excessive NO amplifies inflammatory signaling and cytokine release, its inhibition reinforces the anti-inflammatory profile of the extract (Dandia and Tayalia 2021; Van Broeckhoven et al. 2022). Additionally, HECr reduced IL-1β and TNF-α while increasing IL-13, suggesting a shift in macrophage polarization from a pro-inflammatory (M1) to an anti-inflammatory (M2) phenotype.

Vascular alterations are among the earliest events in the inflammatory response, characterized by vasodilation and increased vascular permeability leading to edema formation (Murphy 2013; Brain and Williams 1985). The reduction in Evans blue extravasation observed after HECr treatment suggests modulation of mediators involved in these vascular changes (Tomisawa and Sato 1973; Lindner and Heinle 1982). Vascular alterations facilitate leukocyte migration to inflamed tissues, where macrophages and other immune cells release cytokines and inflammatory mediators that regulate the recruitment of additional leukocytes, particularly neutrophils (Fujiwara and Kobayashi 2005; Kaplanski et al. 2003).

In the LPS-induced peritonitis model, HECr markedly reduced leukocyte recruitment, especially neutrophils, which are key cells in the acute inflammatory response (Almeida et al. 2016; McDonald et al. 2010). This effect was accompanied by reduced levels of IL-1β and TNF-α and increased anti-inflammatory cytokines, supporting the regulation of inflammatory pathways (Mosser, 2008). In vitro findings, including the inhibition of NO production and increased IL-13, further suggest that HECr promotes a shift toward an anti-inflammatory macrophage profile (M2), reinforcing its therapeutic potential (Dandia and Tayalia 2021; Van Broeckhoven et al. 2022).

In the present study, phytochemical analysis of HECr revealed phenolic acids, hydrolyzable tannins, flavonoids, and quinic acid, a non-phenolic organic acid. Arunachalam et al. (2019) also reported the presence of additional flavonoids and phenolic compounds in this extract, including gallic acid, kaempferol, morin, myricetin, and rutin. These compounds are known for their anti-inflammatory activity: phenolics such as ellagic acid and galloyl derivatives modulate inflammatory mediators and oxidative stress (Larrosa et al. 2010; Ueda et al. 2011), while flavonoids like quercetin, myricetin, and luteolin inhibit pro-inflammatory cytokines and NF-κB signaling (Li et al. 2016; Aziz et al. 2018). Thus, the observed anti-inflammatory effects may be linked to these phenolic compounds, flavonoids, and quinic acid.

The anti-inflammatory effect correlates with the compounds in Table 1, supported by the total ion chromatogram (Fig. 1) and literature on the xylopodium of *C. regium* (Solon et al. 2012; Arunachalan et al. 2019). These compounds are mainly polyphenols, including flavonoids (flavonols and flavones) and hydrolyzable tannins (gallotannins and ellagitannins). Myricetin-3-O-β-D-galactopyranoside (**9**) showed the highest detector intensity, defining the HECr profile alongside quinic acid (**1**) and quercetin-3-O-rhamnoside (**8**). Although major constituents largely drive the biological effect, the activity likely results from synergism with less abundant compounds. Peaks **4**,** 5** and **6**, and peaks **2** and **10** (ellagic acid derivatives), despite lower intensity, generate fragments at 169 (gallic acid) and 301 (ellagic acid). These metabolites exhibit high catalytic affinity and free radical stabilization, complementing the action of polyphenols in *Cochlospermum* species (Solon et al. 2012; Galvão et al. 2023).

The presence of constituents with low absolute intensity, **11** and **12**, reinforces that the chemical profile of the HECr is saturated with polyphenols of different polarities, ensuring a multipotential protection network. This structural evidence, added to the absence of significant signals of other secondary metabolites in the total ion chromatogram, consolidates phenolics as the determinants of bioactivity (Ahmad et al. 2021).

From a mechanistic point of view, the distinction between metabolites lies in the nature of their interactions. Unlike terpenes and steroids (Nunes et al. 2020), which have lipophilic hydrocarbon structures and act non-specifically in the lipid bilayer, the phenolic compounds identified by UHPLC-ESI-IT-MSⁿ have multiple hydroxyl groups (-OH). As demonstrated by fragmentation patterns (neutral loss of hexoses and cleavages of the benzopyr nucleus), these compounds establish selective interaction bridges and π-stacking interactions at the active sites of COX-2 and 5-LOX enzymes (Ahmad et al. 2021).

This observation is corroborated by the literature, which reports high concentrations of phenolic compounds (1,443.04 mg gallic acid equivalents (GAE)/g) and flavonoids (708.75 mg quercetin equivalents (QE)/g) for the *C. regium* extract (Pedroso et al. 2019). This phytochemical density, combined with the consistency of the yield data and the UHPLC analysis that identifies flavonols as the main constituents (Arunachalam et al. 2019), securely establishes the main profile of the extract. Thus, the UHPLC-ESI-IT-MSⁿ and DFI-ESI-IT-MSⁿ data presented confirm that polyphenols are potentially determinants of the related bioactivity.

## Conclusion

HECr exhibits consistent anti-inflammatory activity through modulation of vascular events, leukocyte recruitment, cytokine balance, and NO production, with low cytotoxicity. These effects are likely associated with secondary metabolites, particularly polyphenols and quinic acid. The ethnopharmacological relevance of *C. regium* in Brazil is reinforced by the present findings and warrants further investigation as a promising source for the development of phytotherapeutic agents.

## Data Availability

All data generated or analyzed during this study are included in this article.
